# Development of a Non-invasive Deep Brain Stimulator With Precise Positioning and Real-Time Monitoring of Bioimpedance

**DOI:** 10.3389/fninf.2020.574189

**Published:** 2020-12-08

**Authors:** Heng Wang, Zhongyan Shi, Weiqian Sun, Jianxu Zhang, Jing Wang, Yue Shi, Ruoshui Yang, Chunlin Li, Duanduan Chen, Jinglong Wu, Guo Gongyao, Yifei Xu

**Affiliations:** ^1^School of Mechatronic Engineering, Beijing Institute of Technology, Beijing, China; ^2^School of Life Science, Beijing Institute of Technology, Beijing, China; ^3^Department of Health Management, Aerospace Center Hospital, Peking University Aerospace School of Clinical Medicine, Beijing, China; ^4^Beijing Big-IQ Medical Equipment Co., Ltd., Beijing, China; ^5^School of Biomedical Engineering, Capital Medical University, Beijing, China; ^6^Graduate School of Interdisciplinary Science and Engineering in Health Systems, Okayama University, Okayama, Japan

**Keywords:** electrical stimulation, temporally interfering, finite element method, simulation, mouse

## Abstract

Methods by which to achieve non-invasive deep brain stimulation via temporally interfering with electric fields have been proposed, but the precision of the positioning of the stimulation and the reliability and stability of the outputs require improvement. In this study, a temporally interfering electrical stimulator was developed based on a neuromodulation technique using the interference modulation waveform produced by several high-frequency electrical stimuli to treat neurodegenerative diseases. The device and auxiliary software constitute a non-invasive neuromodulation system. The technical problems related to the multichannel high-precision output of the device were solved by an analog phase accumulator and a special driving circuit to reduce crosstalk. The function of measuring bioimpedance in real time was integrated into the stimulator to improve effectiveness. Finite element simulation and phantom measurements were performed to find the functional relations among the target coordinates, current ratio, and electrode position in the simplified model. Then, an appropriate approach was proposed to find electrode configurations for desired target locations in a detailed and realistic mouse model. A mouse validation experiment was carried out under the guidance of a simulation, and the reliability and positioning accuracy of temporally interfering electric stimulators were verified. Stimulator improvement and precision positioning solutions promise opportunities for further studies of temporally interfering electrical stimulation.

## Introduction

Considering the challenges associated with an aging society, brain diseases have increasingly serious negative effects on human life (Cole and Franke, 2017). Continued investigation into therapies for brain diseases should be encouraged to expand indications and improve effectiveness (Buss et al., 2019). As a typical neurosurgical procedure, DBS has been used to cure abnormal neuronal firing patterns that result from certain diseases, such as Parkinson's disease, essential tremors, and dystonia (Flora et al., 2010; Miocinovic et al., 2013). However, careful wound care and personal hygiene are needed to protect DBS hardware and to avoid additional negative impacts after the surgery (Umemura et al., 2003; Blomstedt and Hariz, 2006; Batra et al., 2016). Studies on non-invasive brain stimulation that does not require built-in hardware are accumulating rapidly. Transcranial direct current stimulation (tDCS) and transcranial alternating current stimulation (tACS) are common non-invasive tools that use weak electric currents to painlessly and non-invasively regulate human neural activity and are widely employed in many research areas (Ali et al., 2013; Schulz et al., 2013; Tavakoli and Kyongsik, 2017). Due to the characteristics of current transmission, these non-invasive electrical stimulation methods have a qualified stimulation effect on superficial brain areas, but it is difficult to reach deep targets accurately via these techniques.

In 2017, Cell magazine reported a temporally interfering electrical stimulation technology using multiple high-frequency alternating currents to recruit neural firing (Grossman et al., 2017). Compared with common non-invasive electrical stimulation, time-interfering electrical stimulation can directly reach deep brain regions without affecting shallow brain regions. However, as a new technology, the stimulator needs to be improved to help researchers make it more efficient and convenient to use for temporally interfering electrical stimulation. The stimulus effect is based on the envelope modulation of the electric field, and the envelope will appear seriously distorted or too small if one of the loads is too large. Therefore, for the stimulator, real-time measurements of biological impedance between electrodes and warnings of potential overloading are necessary. In addition, the positional accuracy of temporally interfering electrical stimulation is jointly determined by the electrode position and the current amplitude ratio. An existing study has described methods by which to move the position of the target of temporally interfering electrical stimulation, and a helpful rule for adjusting the location of the electrical stimulation target was proposed—the wider the electrode spacing is, the deeper the stimulation target depth (Grossman et al., 2017). However, an accurate positioning scheme for specific targets is lacking, and the qualitative rule cannot be directly used to calculate the location of the electrode. Several studies have used arrays of scalp electrodes, with each electrode optimized to target a desired location in the human brain (Huang and Parra, 2019). The electrode array optimization method may not be appropriate in a mouse model because of the small size of the mouse head and the fact that the electrodes cannot be shrunken indefinitely. The number of electrodes in the preset electrode array is not large enough.

In this study, a powerful temporally interfering stimulator was developed. In terms of accuracy, we did the following work. First, we solved the problem of crosstalk between channels through a circuit design and improved the accuracy of each circuit stimulation signal. Second, to avoid the waveform distortion and amplitude decrease caused by excessive impedance, a bioimpedance measurement module was designed. Due to these two developments, the stimulator has advantages in terms of precision. To form modulation envelope waves with accurate frequency characteristics through the interference of kHz differential frequency currents, the kHz-level sinusoidal electrical stimulation signal of the electrical stimulator must have ultrahigh parameter control accuracy. The device that we designed adopts graphical user interface control and integrates a bioimpedance measurement function. The amplitude, frequency, sinusoidal phase, and transition time can be precisely controlled to ensure the stability and controllability of the complex intracranial electric field interference. Based on the idea that biological impedance varies with the excitation frequency (Stroud et al., 1995), the appropriate carrier frequency can be selected to achieve a small current loss.

We conducted a simulation analysis and a phantom measurement proof to study the functional relationships among the target location, the electrode location, and the current ratio in temporally interfering stimulation. The functional relationship between the electrode spacing and the stimulus depth was fitted to locate the longitudinal coordinate of the target. In addition, the functional relationship between the amplitude ratio of the currents and the transverse coordinate of the target was fitted to assist in locating the transverse target coordinate. By solving these functions, the electrode arrangement can be directly determined according to the coordinates of the target. Then, we formed a set of feasible schemes by which to achieve accurate positioning in a simplified and realistic mouse model. Based on the positioning functions and the mouse model simulation, the auxiliary software was designed to help target the desired location in the mouse brain. In this way, it is convenient to use the temporally interfering stimulation system in mouse experiments for those who are not interested in modeling and simulation. Finally, a small region of the mouse motor cortex associated with shoulder movement was successfully located and activated in the mouse experiment. The experiments demonstrated that the electrical stimulator could effectively modulate mouse neurons by enveloping the electric field, and the localization accuracy was as expected.

## Materials and Methods

The experimental protocol was approved by the ethics committee of Capital Medical University and was in accordance with the Declaration of Helsinki.

### Implementation of the Temporally Interfering Stimulation System

**Figure 1:**
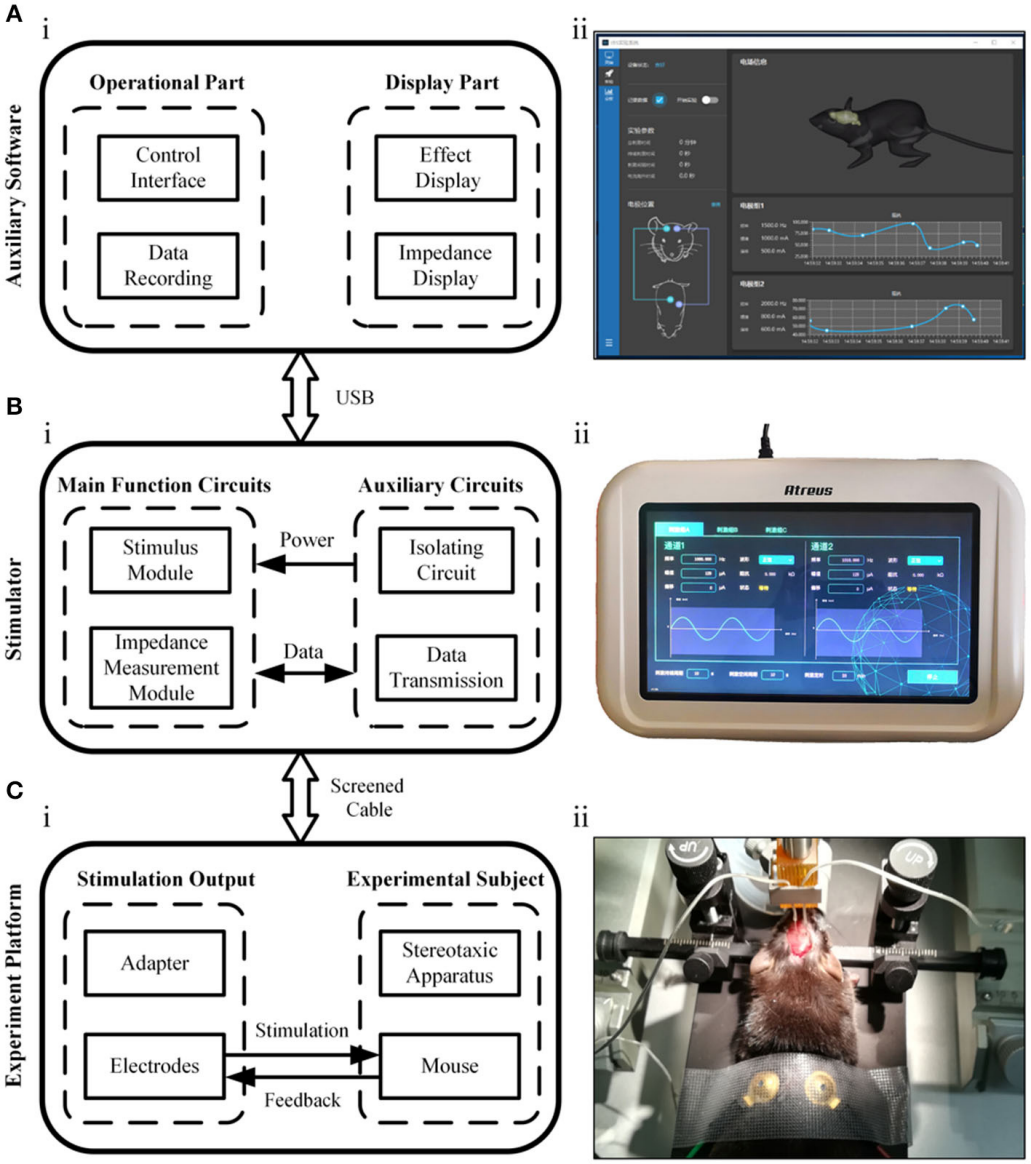
Illustration of the neuromodulation system. **(A)** The auxiliary software. **(B)** The electrical stimulator. **(C)** The experimental platform.

**Figure 2:**
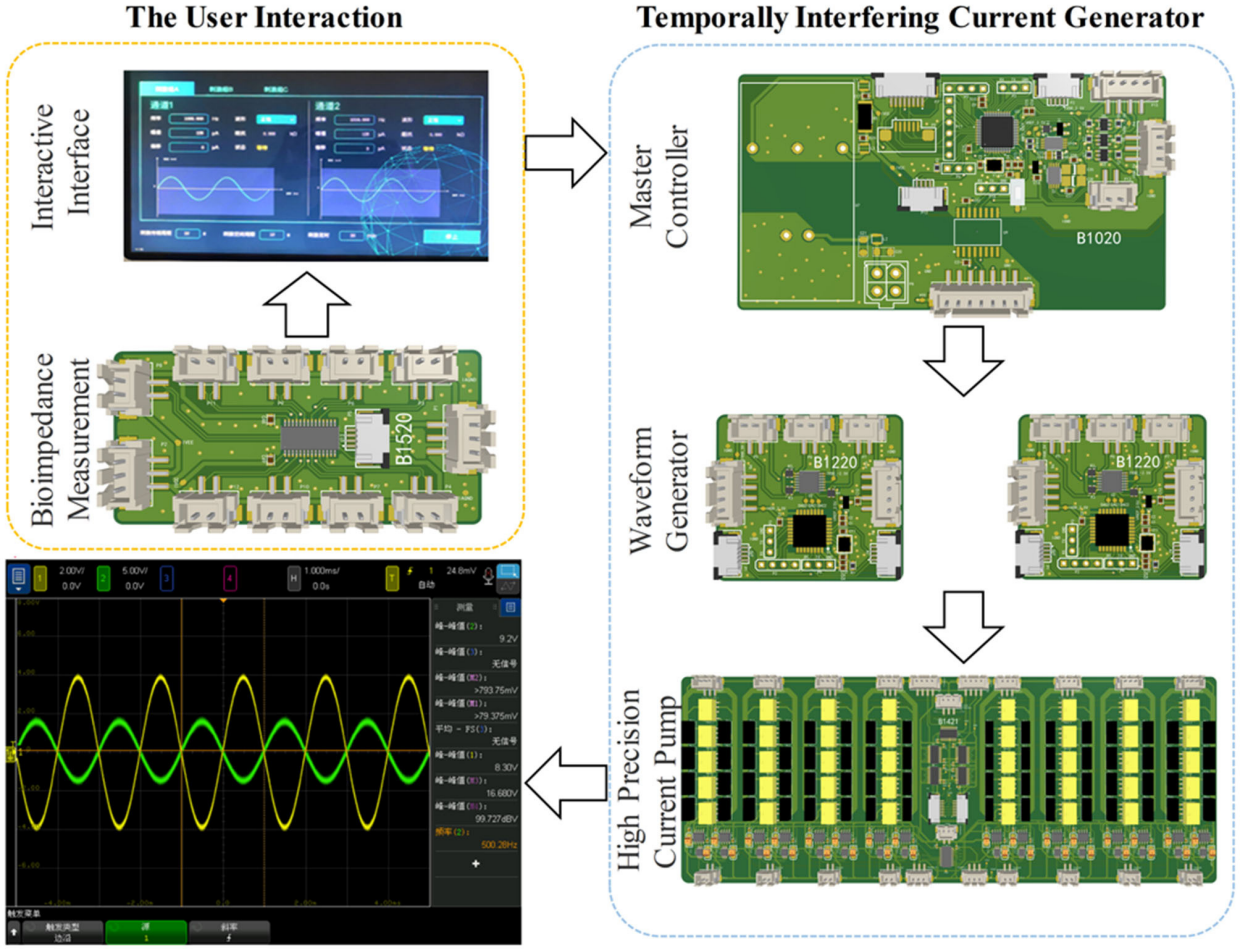
Hardware modular architecture.

Figure 1 shows the conceptual block diagram and photos of the temporally interfering neuromodulation system, including the auxiliary software, electrical stimulator, and experimental platform. The auxiliary software can control the stimulus parameters, record experimental data, and assist in target navigation (Figure 1A). The stimulation parameters, such as the frequency, amplitude, and offset, can also be set on the touch panel of the stimulator. There are two main functions of the stimulator (Figure 1B). One is to provide multichannel, high-precision, and high-frequency output. The other is to measure the bioimpedance synchronously between the stimulation electrodes. When the system works, two sets of high-frequency electrical stimuli are applied to mice via the electrodes, and feedback signals are concurrently collected for impedance measurement (Figure 1C). The electrical stimulator has a modular design, with extra functionality loaded on demand (Figure 2).

#### Implementation of High-Precision Output

The temporally interfering electrical stimulation scheme in this study is based on two high-frequency, accurate alternating currents. In this study, an analog phase accumulator was used to achieve a high-precision sine wave. Since STM32 MCU has no special phase accumulator digital-to-analog converter (DAC), the principle of the phase accumulator can be simulated by software. This process was performed by a system that contained the following main components: a numerically controlled oscillator (NCO), a frequency and phase modulator, SIN ROM, a digital-to-analog converter, and a regulator. The DAC was configured for single-ended operation. The simulation process was achieved by continuously updating the output data to the direct memory access (DMA). After optimization, the test could be performed in 1,000 ns for each output but in 522 ns for each data-generation instance. Up to 52.2% of the CPU was utilized. When using two channels, the output frequency could be reduced to 250 kHz. To prevent the user from reducing the frequency too much, all subordinate machines were cross-connected; that is, the output of the original 1–2 channels was provided by subordinate machine 1, whose output was jointly provided by subordinate machines 1 and 4 after using this algorithm.

#### Implementation of Bioimpedance Measurements

The biologically complex impedance measurement technique used in this study collects the potential signal produced by the corresponding frequency stimulation. The measured bioimpedance was the sum of the tissue impedance and the electrode contact impedance. The process used to implement the bioimpedance measurement function was as follows. First, the electrical signal output module of the electric stimulator was programmed to provide a weak sinusoidal signal to the electrode group attached to the subject, and the feedback signal was collected at the same time. Then, discrete Fourier transform arithmetic was used to calculate the real and imaginary parts of the signal (Yang et al., 2017). The real part R and imaginary part I can be converted into amplitude and phase information by Equations (1) and (2). Using this method, the device can measure the impedance of 100 Ω-10 M Ω values and achieve a system accuracy as high as 0.5% (Analog Devices Inc, 2005–2011). The function of bioimpedance measurement helps ensure the accuracy of the waveform in the electrical stimulation experiment, thus ensuring the effect of the electrical stimulation. The circuit design details are shown in the Supplementary Figure 2.

(1) $$\begin{array}{l} Magnitude=\sqrt{R^{2}+I^{2}} \end{array}$$

(2) $$\begin{array}{l} \text{Phase}=Tan^{-1}\left(I/R\right) \end{array}$$

#### Inhibition of Crosstalk Between Channels

The output circuit of the temporally interfering electrical stimulator consists of three parts: an amplifying circuit, a reverse dual-current pump, and a limiter circuit. In the temporal interfering electrical stimulation, the current flows not only from the output electrode to the reference electrode but also from the electrodes of the other channels, which results in serious current crosstalk between channels. To maintain the independence of the channel outputs, anti-phase current drive technology was used as shown in the Supplementary Figure 3. Each channel contains two current sources that remain in the opposite phase. In this way, the current between the channels can be balanced to eliminate crosstalk.

### Electrical Stimulator Test

First, a resin phantom was made to test the output performance of the electrical stimulator. The phantom was a 50 mm-diameter cylindrical container filled with a solution with a conductivity set to ~0.333 S m^−1^, and the bottom was covered with holes as fixing points for electrodes. The crosstalk between channels was obtained by analyzing the potential data from the stimulating electrodes (Figures 4Ai,Bi). Second, the load capacity of the electrical stimulator was tested with resistors. We selected 0.5, 1, 1.5, and 2 mA as the current output amplitudes and used gradually increasing resistance as the load. In this process, an ammeter was used to measure the real current output. The critical load values were also recorded. To test the bioimpedance measurement function of the device, we performed three types of load tests—resistance, resistance–capacitance, and mouse—and measured the impedance changes under different excitation frequencies (Figures 5A–C). The first two loads were standard models, and the impedance changes followed physical characteristics that made it easy to determine whether the device measurements were accurate. Third, by comparing the simulated distribution of the electric field envelope amplitude with the measured distribution, the reliability of the temporally interfering electrical stimulator could be confirmed. To obtain the simulated envelope amplitude distribution map, a two-dimensional 50 mm-diameter circular model containing 1,222 grids and 652 nodes was established (Figure 6Ai). The model has been validated for grid independence. The material of the model was set to be uniform and isotropic, and the conductivity was set to 0.333 S m^−1^, which was consistent with the phantom. Two pairs of electrodes were symmetrically placed on the periphery of the model. A sinusoidal waveform current (0.5 mA, 1 kHz) was applied to the electrodes on the left. Another current (0.5 mA, 1.01 kHz) was applied on the right. The measured electric field strength was calculated by Equation (3) to obtain the electric field envelope amplitude.

(3) $$\begin{array}{l} |{\vec{E}}_{AM}\left(\vec{n},\vec{r}\right)|=||\left({\vec{E}}_{1}\left(\vec{r}\right)+{\vec{E}}_{2}\left(\vec{r}\right)\right)\cdot \vec{n}|-\\ \left|\left({\vec{E}}_{1}\left(\vec{r}\right)-{\vec{E}}_{2}\left(\vec{r}\right)\right)\cdot \vec{n}|\right| \end{array}$$

**E1** and **E2** represent the fields generated by the first and the second electrode pairs, respectively; *n* is a unit vector along the direction studied; and *r* represents the location. Then, we drew the simulated distribution map of the envelope amplitude of the electric field intensity.

To obtain the measured envelope amplitude distribution map of electric field intensity, a phantom and an oscilloscope were used (Figure 6B), an alternating current (0.5 mA, 1 kHz) was applied to the left two electrodes. Another current (0.5 mA, 1.01 kHz) was applied on the right. The potential difference ΔV of every two adjacent positions was measured by an oscilloscope, and the electric field intensity E was calculated by equation E(t) = V(t)/S, where S is the distance between the two electrodes inserted at the tested positions. We used the Hilbert transform to process the data of the electric field intensity along the X and Y directions and calculated the envelope amplitude. Then, the measured distribution map of the envelope amplitude could be drawn.

### Accurate Control of the Electrical Stimulation Target

A study of electrical stimulation simulation showed that CSF had a shunt effect on the stimulating current, and the higher the conductivity of CSF, the more obvious the shunt effect was. However, there was no gross change in the current flow patterns through the brain (Jiang et al., 2020). Therefore, even with CSF shunting, electrical stimulation could still reach the target in the brain and showed little effect on the intensity distribution of stimulation in the brain regions. Considering that the CSF layer in mice is too thin to be modeled, we constructed a layered finite element model containing 56,000 grids, including the scalp, skull, and brain, based on the MRI and CT data of the mouse (Figure 8A). The material of each layer was set to be uniform and isotropic, and the conductivity was set to 0.333, 0.0083, and 0.333 S m^−1^, corresponding to the scalp, skull, and brain, respectively (Grossman et al., 2017). To reduce the computational load, a layered elliptic cylinder model was constructed according to the shape of the coronal plane of the mouse head (Figure 7). The major axis of the ellipse was 2a, the minor axis was 2b, and the thickness of the first layer was 0.44 mm, as in the mouse skull. Afterward, the layered elliptic cylinder model was further simplified into a layered circular cylinder model by keeping the curvature radius unchanged, with the radius R = a^2/*b*^. Taking the radial electric field stimulation as the most important part, we analyzed the distribution diagram of the electric field enveloping intensity in this direction. We conducted multiple sets of simulations to find the relationship between the target depth and the electrode distance of the electrical stimulation in an ideal layered circular cylinder model. In addition, the transverse coordinates of the target were expected to be correlated with the electrode position. The results of the simplified model were applied to the individual mouse model to verify the validity of the rule. The simulation results were used in the software of a temporally interfering electrical stimulation system, with which we predicted and adjusted the electrode positions to accurately stimulate the target.

### Mouse Experiment

**Figure 3:**
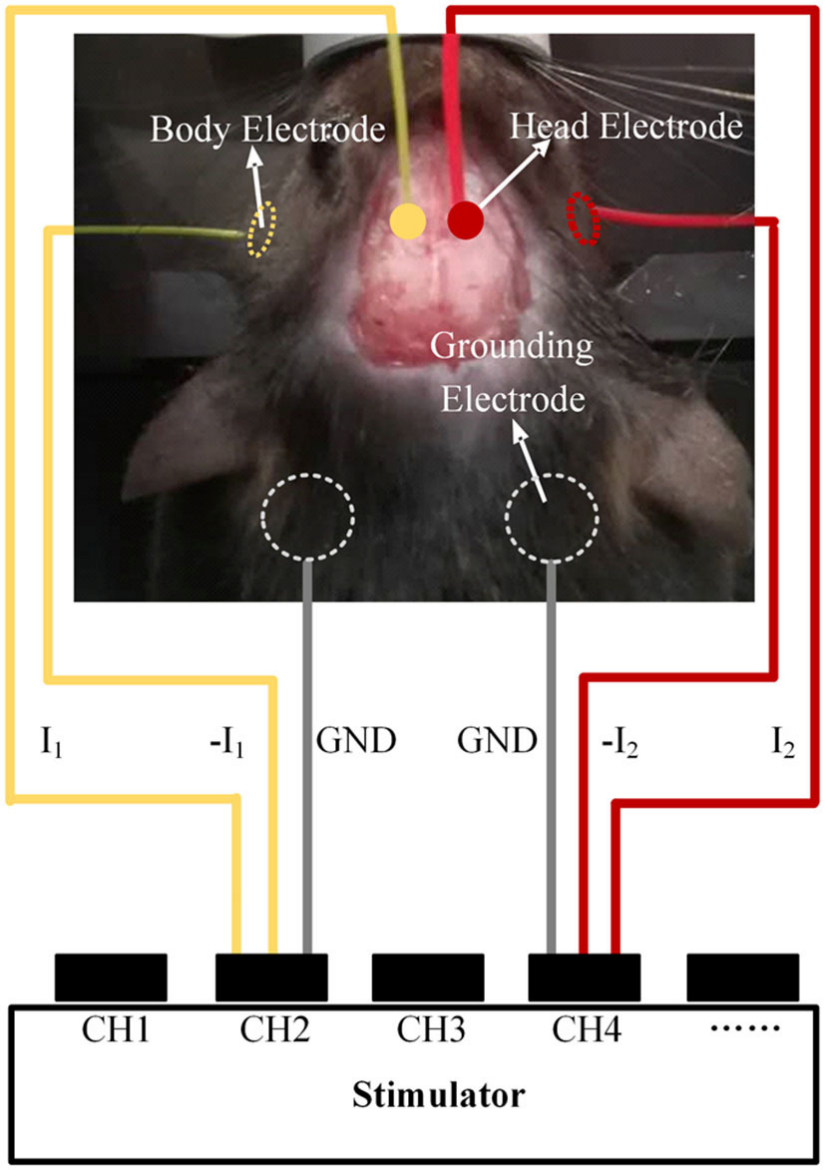
Schematic diagram of the animal experiments.

Ten male C57BL/6 mice were subjected to electrical stimulation in the motor cortex to activate neurons to cause evoked movements. The motor cortex area related to the shoulder is ~1 × 1 mm, and we selected it as a target of temporally interfering electrical stimulation (Tennant et al., 2011). The target position relative to the bregma was AP −0.5 mm and ML 0.75 mm. Overall swinging of the mouse front paw should be observed as the target phenomenon. To prepare for the experiment, the mice were anesthetized with isoflurane, and surgery was performed to expose the skull and bregma. In addition, the cheeks of the mice were shaved. The electrical stimulation experiment was performed immediately after preparation, and the mice were maintained under continuous anesthesia with 1–1.5% (vol/vol −1) isoflurane in oxygen. Two 1 mm-diameter head electrodes with conductive paste were attached to the surface of the skull. Two 2 mm-diameter body electrodes with conductive gel were attached to the cheek of the mouse, spaced ~1 cm apart. Two 5 mm-diameter grounding electrodes were placed on the shaved chest, spaced ~0.8 cm apart. The electrode configurations were determined through simulation in the mouse model. The two head electrodes were set mediolaterally at −0.55 and 2.05 mm and anteroposteriorly at −0.5 mm relative to the bregma. Each of the two kHz alternating currents were applied to the mouse simultaneously through the head electrode and the body electrode connected to the electrical stimulator (Figure 3).

First, we performed a stimulation experiment with alternating currents of I1 and I2 (1 kHz 50 μA, 1.002 kHz 50 μA). If a 2 Hz overall swing of the contralateral forepaw was not observed, the sum of the current amplitudes I1 + I2 was increased by a gradient of 50 μA. In the process, the positioning of the target could also be finetuned by adjusting the proportion between the two currents. Then, we recorded the lowest I1 + I2 that evoked contralateral forepaw movement. Movement of the ipsilateral forepaw during stimulation was also recorded. Finally, we changed the frequency of I2 to 1.005 kHz and 1 kHz and recorded the experimental phenomena.

## Results

### Performance Test of the Electrical Stimulator

#### Implementation of High-Precision Output

**Figure 4:**
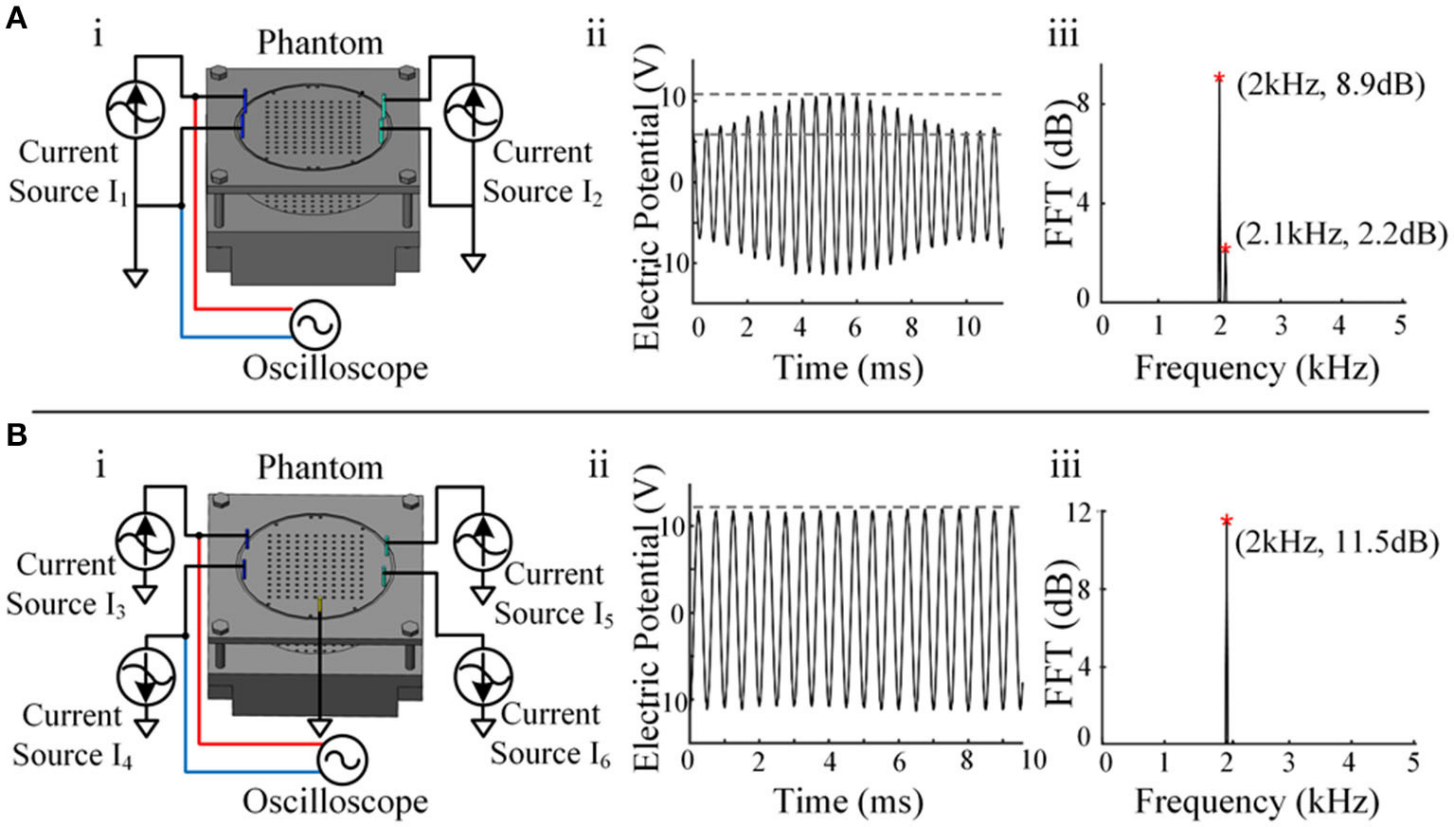
Crosstalk testing between channels. **(A)** i, Schematic diagram of a single-current source drive. ii, The waveform between the two electrodes of a single-current source output channel. iii, Spectrum analysis result of a single-current source output channel. **(B)** i, Schematic diagram of the reverse dual-current source drive. ii, The waveform between the two electrodes of the dual-current source output channel. iii, Spectrum analysis result of the dual-current source output channel.

Four electrodes were applied in the phantom (Figure 4Ai), and an electrical stimulator channel was used to output 2 kHz on the left and 2.1 kHz on the right via another channel. The waveform shows the interference state of two sine waves between the two electrodes (Figure 4Aii). After spectral analysis, the two components of 2 and 2.1 kHz could be clearly observed, indicating that there was mutual interference between the two channels (Figure 4Aiii).

The waveform between the two electrodes presented a relatively standard sine wave (Figure 4Bii), and only the composition of 2 kHz was seen after spectral analysis, indicating that the crosstalk between the two channels was reduced significantly (Figure 4Biii). In this way, channel-to-channel isolation for high-quality current output was achieved. A single-channel current source drive can remain stable when stimulated by a single channel, but serious crosstalk occurs when it is stimulated by dual channels simultaneously, as temporally interfering electrical stimulation occurs. Reverse dual-current pump drive technology successfully solved the crosstalk problem.

#### Output Performance Testing

**Figure 5:**
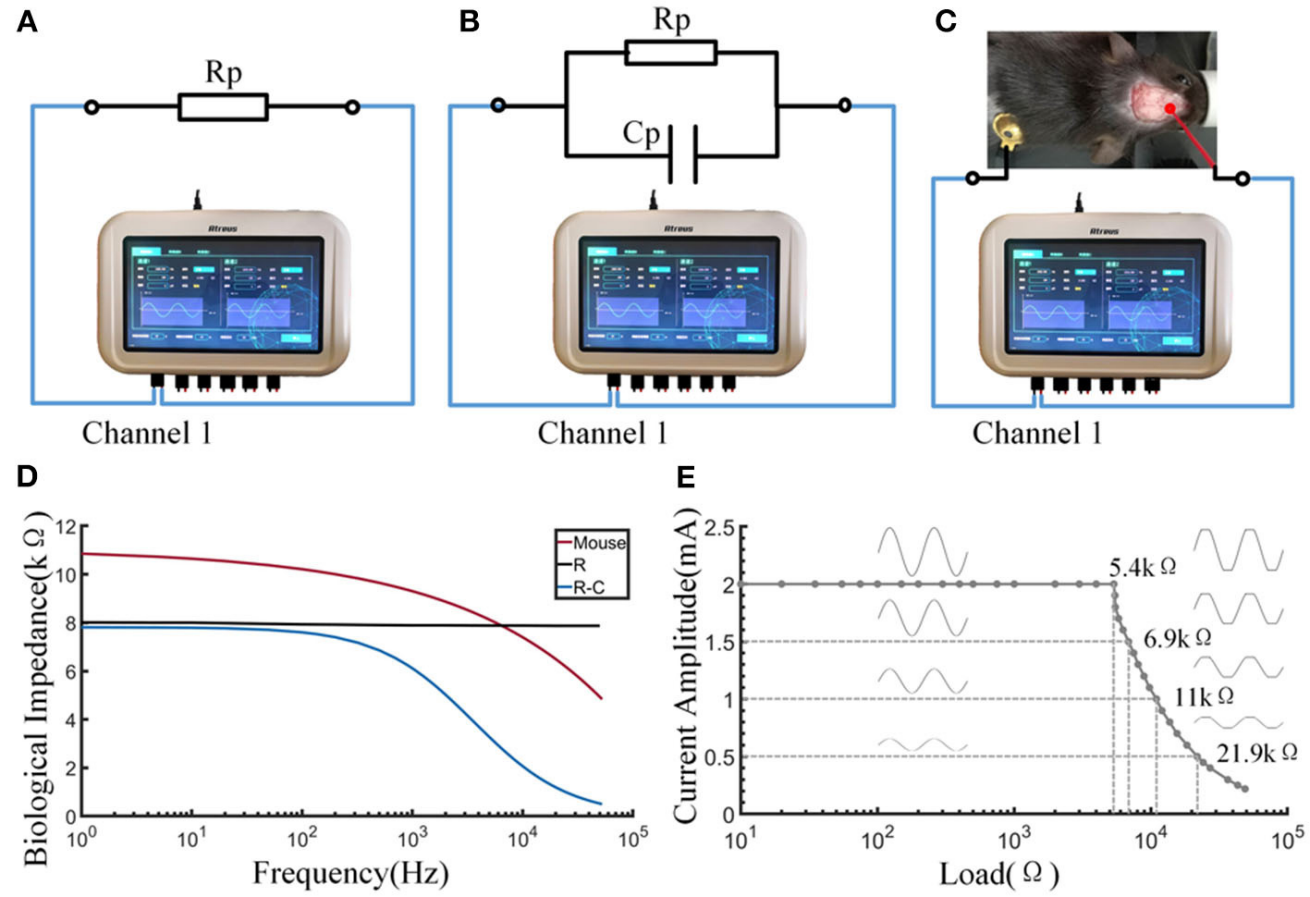
Output and impedance measurement performance of the stimulator. **(A)** Schematic diagram of measuring the resistance by the stimulator. **(B)** Schematic diagram of measuring the resistance-capacitance by the stimulator. **(C)** Schematic diagram of measuring the bioimpedance of a mouse by the stimulator. **(D)** The curve of impedance by excitation frequency. **(E)** The load curve of the stimulator.

The output waveform of the electric stimulator produced accurate signals when the load did not exceed the range (Figure 5E). The electrical stimulator could provide a standard sinusoidal signal of 2 mA amplitude within the load range of 0–5.4 kΩ. Waveform distortion occurred if the load was exceeded, as shown by a series of tests with various resistances. The load capacity decreased as the set current increased. In terms of the biological impedance measurement function of the device, three types of impedance—resistance, resistance-capacitance, and mouse body—were tested (Figures 5A–C). Electrodes were placed on the skull and body of the mice, which was consistent with the electrical stimulation. The impedance of 8 kΩ resistance did not change with the excitation frequency, and the impedance of the resistor-capacitor load decreased with increasing excitation frequency and closely matched the result of the calculation formula Z = R/(1+j × R × ω × C). This result indicates that the biological impedance measurement function of the equipment is qualified. Impedance measurements in mice showed that impedance values decreased as the frequency increased, somewhat similar to the resistance-capacitance model (Figure 5D).

#### Comparison of Simulation Calculations and Stimulation Measurements

**Figure 6:**
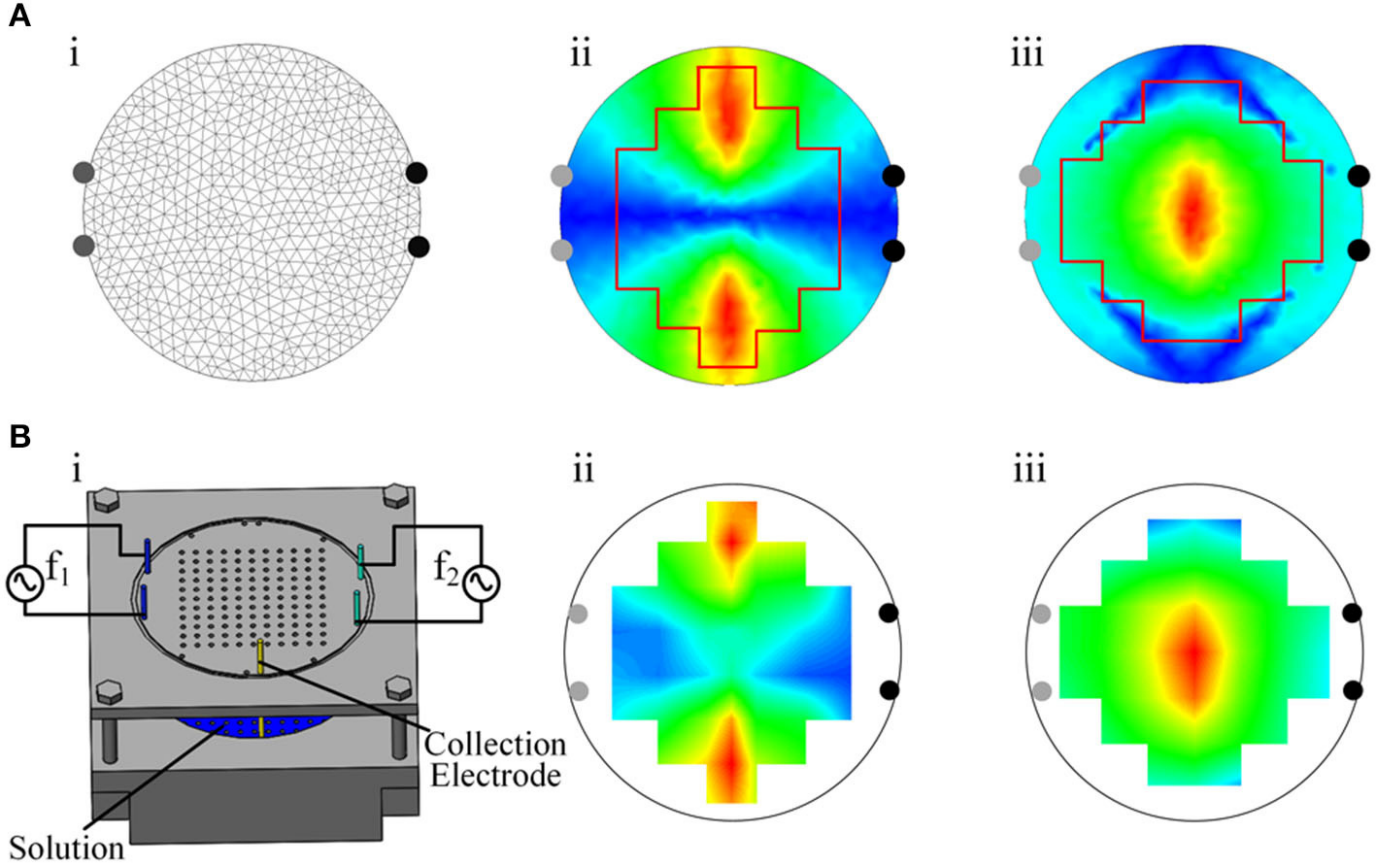
Measured and simulated distribution of the envelope amplitude. **(A)** i, Two-dimensional finite element model. ii, Simulated distribution of the envelope amplitude of the electric field intensity in the X-axis direction. iii, Simulated distribution of the envelope amplitude of the electric field intensity in the Y-axis direction. **(B)** i, Phantom. ii, Measured distribution of the envelope amplitude of the electric field intensity in the X-axis direction. iii, Measured distribution of the envelope amplitude of the electric field intensity in the Y-axis direction.

In this study, we simulated the envelope amplitude on a 2-dimensional circular model and compared the distribution map with the phantom-measured result. The simulated and measured results were based on the model and the phantom (Figures 6Ai,Bi). The X-axis direction and the Y-axis direction were chosen as the focus directions of the electric field intensity. Upon comparing the measured distribution map of the envelope amplitude of the electric field (Figures 6Aii,iii) with the simulated one (Figures 6Bii,iii), similar distributions and the same legend were found. The unification of the two types of envelope amplitude distributions demonstrated the accuracy of the electrical stimulator output. This result confirmed the reliability of the electrical stimulator from a comprehensive perspective.

### Mouse Experiment

#### Simulation Prediction of Electrode Positions

**Figure 7:**
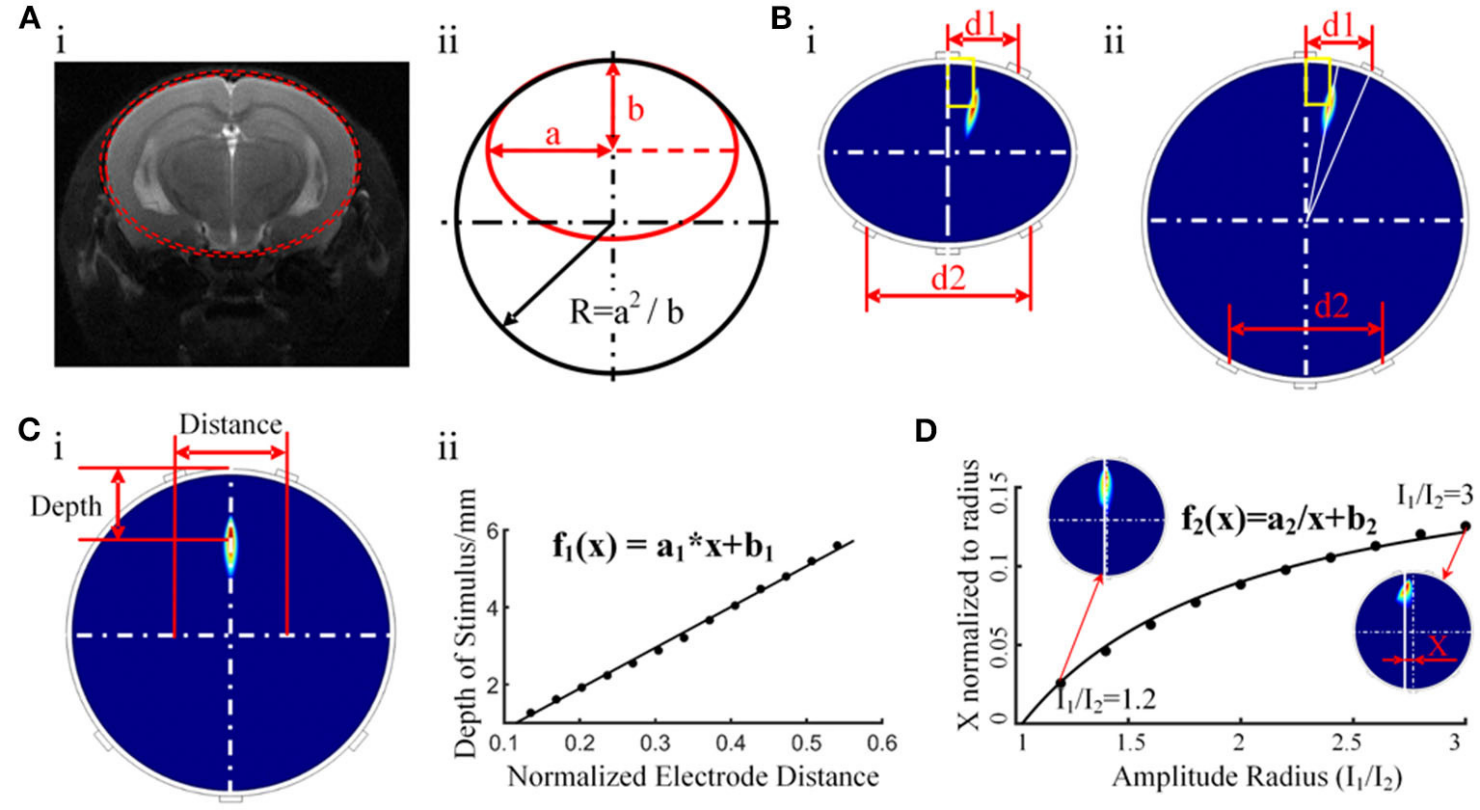
Initial target positioning via a simplified columnar model. **(A)** i, The ellipse model constructed with MRI data. ii, Further simplified circular model. **(B)** i, Distribution of the electric field envelope amplitude in the elliptic cylinder model. ii, Distribution of the electric field envelope amplitude in the circular cylinder model. **(C)** Functional relationship between the depth of the temporally interfering stimulation target and the distance between the head electrodes. **(D)** Relationship between the amplitude ratio (I1/I2) and the peak envelope amplitude position.

The motor cortex target position of the mouse experiment relative to the bregma was AP −0.5 mm and ML 0.75 mm, and the depth was 0.44 + 0.8 mm (the thickness of the mouse skull was 0.44 mm, and 0.8 mm corresponded to the mid-layer to deep-layer V) (Tennant et al., 2011; Lapchak et al., 2015). We simplified the coronal plane of mice into a two-layer elliptic model and then further simplified it into a circular model by keeping the maximum curvature radius unchanged and radius R = a^2/*b*^ (Figure 7A). The major axis of the ellipse was 2a, the minor axis was 2b, and the radius of the circular model was R. According to the MRI images of the mouse brain, we measured the coronal section at AP 0.5 mm and determined that a = 4.1 mm, b = 3.6 mm, and R = 4.7 mm. The thickness of the first layer was 0.44 mm, which was the same as that of the mouse skull, and the conductivity was 0.0083 S m^−1^. The second layer represented the brain, with a conductivity of 0.333 S m^−1^. The simulated targets of the elliptic and circular models were the same as the same electrode position and current parameters (Figure 7B). In addition, when two current amplitudes are the same, the radial electric field envelope distribution will be in the middle of the two nearest electrodes. In the circular model, there is a linear relationship between the depth of the target of the electrical stimulation and the distance between two relatively close electrodes. The specific functional relationship was established (Figure 7C), with a1 = 0.6343 and b1 = −0.0773. According to the functional relationship, the electrode distance should be 2.6 mm to stimulate a target with a depth of 1.24 mm. Therefore, the two head electrodes should be set mediolaterally at −0.55 mm and anteroposteriorly at −0.5 mm and mediolaterally at 2.05 mm and anteroposteriorly at −0.5 mm relative to the bregma.

In contrast to ordinary electrical stimulation, in which the stimulation target can only be changed by adjusting the electrode position, temporally interfering electrical stimulation can conveniently adjust the coordinates of the stimulation area by changing the amplitude ratio between the two currents. A cylindrical simulation model was built to study the effect of the current amplitude ratio on the location of the peak envelope amplitude area. Upon keeping the electrode position unchanged and adjusting the ratio of the current amplitude, the peak stimulation area moved along the X-axis and maintained a stable position along the Y-axis. Through the function-fitting analysis of the ratio of the current amplitude and peak area position, a function with good coincidence was found (Figure 7D), with a2 = −0.1906 and b2 = 0.1853. Based on this finding, adjusting the current ratio is proposed to control the lateral location of the peak stimulation region. Together with the rule indicating that the longitudinal depth position is controlled by adjusting the spacing of the head electrodes, the function can make target positioning more convenient.

**Figure 8:**
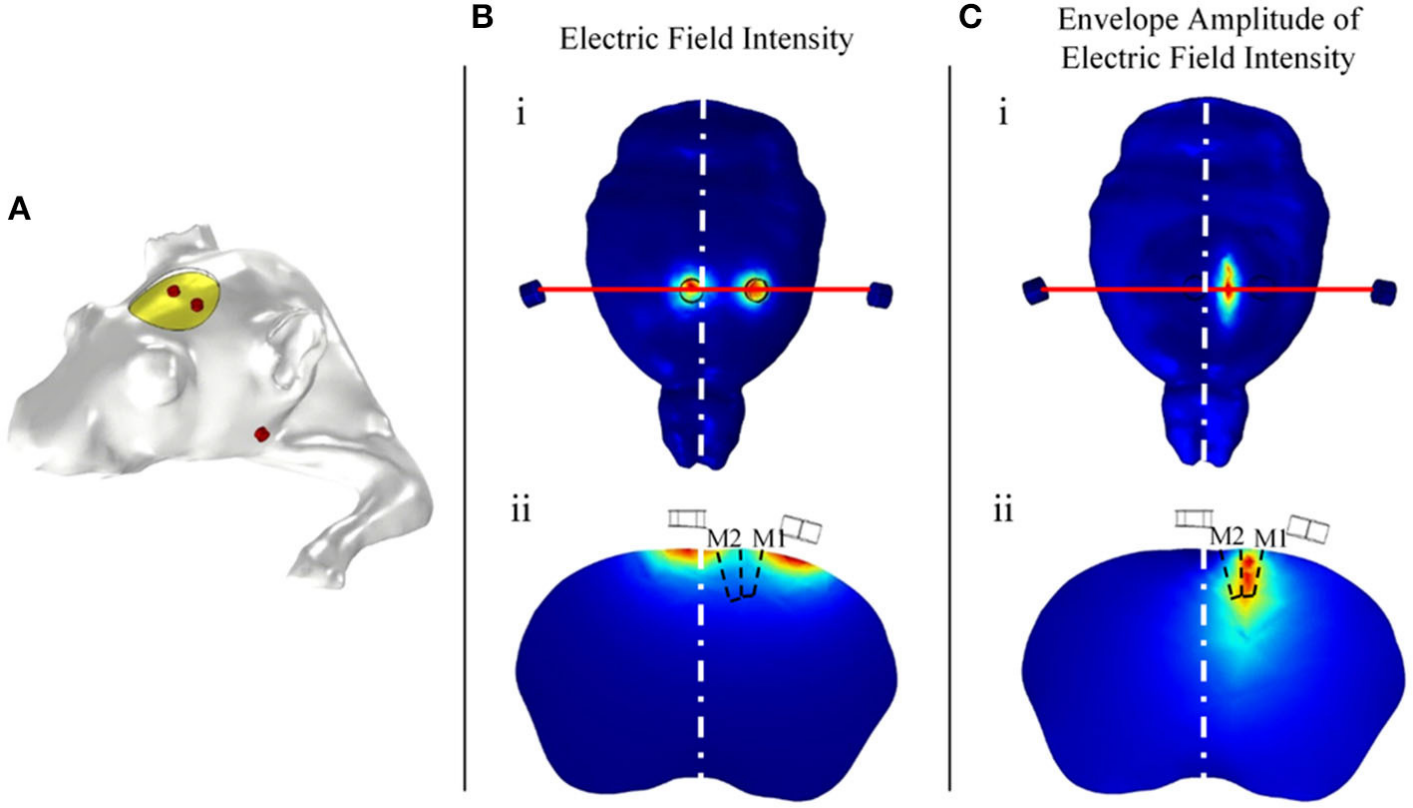
Relationship between the head electrode positions and the distribution of the envelope amplitude. **(A)** Individual simulated mouse model. **(B)** Distribution of the electric field intensity. **(C)** Distribution of the electric field envelope amplitude intensity.

The above simulation results regarding electrode positions in the simplified model require simulation verification in an individualized mouse model. The individualized mouse model uses the mouse bregma as the coordinate origin. According to the simulation results of the simplified model, 1 mm diameter electrodes were placed at positions (2.05, 0, −0.5) and (−0.55, 0, 0.5). Two body electrodes were placed symmetrically on the cheeks of the mice. The simulation results show that the radial electric field envelope amplitude was the largest in the section in which the electrode was placed (Figures 8B,C). The electric field of the electric stimulation under the head electrode was the largest, but the maximum amplitude of the electric field envelope as an effective stimulation occurred in the middle between the head electrodes. The simulation results show that the coordinates of the region with the largest envelope amplitude were (0.71, −1.12, −0.57), with a deviation of 0.15 mm from the target point (0.75, −1.25, 0.5).

#### Mouse Stimulation Experiment

Electrodes were placed according to the simulation result to stimulate the target in a mouse stimulation experiment. I1 (1 kHz) and I2 (1.002 kHz) were supplied by the stimulator. The experimental phenomena and electrical stimulation parameters are shown in Table 1. The result shows that every mouse experienced evoked movements in the forepaw on the contralateral side, and the frequency of the periodic movement was equal to the frequency difference between the two channels. The mouse forepaw moved only as a whole, and smaller joints such as the wrist or elbow did not move. This finding suggests that the electrical stimulator activated mouse neurons and successfully targeted the 1 × 1 mm motor cortex related to shoulder movement. In addition, I1 (1 kHz) and I2 (1.005 kHz) were also used to stimulate the mouse, and higher-frequency evoked movements were observed. However, I1 (1 kHz) and I2 (1 kHz) caused no experimental phenomena.

**Table 1:** Experimental data of mice.

**No**.	**Current amplitude I1/I2 (μA)**	**Whether contralateral forepaw shook**	**Whether ipsilateral forepaw shook**
1	200/150	YES	NO
2	200/200	YES	NO
3	150/150	YES	NO
4	150/150	YES	NO
5	200/150	YES	NO
6	150/150	YES	NO
7	150/150	YES	NO
8	200/200	YES	NO
9	150/200	YES	NO
10	200/200	YES	NO

## Discussion

In this study, we developed a multichannel temporally interfering electrical stimulation system with a target positioning function. The real-time bioimpedance measurement function of the stimulator ensures that the actual stimulation is accurate and as expected. In terms of the accurate target positioning, we constructed individualized mouse models, performed finite element analysis of the electric field, and successfully simplified the simulation using cylinder models of layered ellipses and layered circles. We found the functional relationship between the stimulus depth and the electrode spacing, as well as the relationship between the target abscissa and amplitude ratio. These findings can help users achieve target positioning in mice without the heavy work of modeling and simulation. The mouse experiment showed that the stimulation system could indeed activate mouse neurons, and the accuracy of target localization was satisfactory.

In most studies of tACS, 10–40 Hz and 0.4–1 mA currents are used (Antal et al., 2008; Zaehle et al., 2010; Paulus, 2011). In addition, 140 Hz tACS on the primary motor cortex has been shown to result in nonlinear excitatory modulation of cortical tissue (Moliadze et al., 2012). All these studies used a one-channel electrical stimulator (Version DC-Stimulator-Plus, NeuroConn) to provide stimulation with adjustable frequencies up to 250 Hz. In addition, tRNS can also be considered a type of tACS, with frequencies varying from 0.1 to 640 Hz (Terney et al., 2008). The sampling rate of the electrical stimulator (Version Eldith DC-Stimulator-Plus, NeuroConn) used in this tRNS study was 1,280 samples/s, with frequencies adjustable up to half of the sampling rate, i.e., 640 Hz. These stimulators can only provide low-frequency signals. Later studies found that stimulation at 2 and 5 kHz produced lasting changes in the motor cortical excitability, which was attributed to the modulation of neuronal membrane activity (Herrmann et al., 2013). The kHz stimulation was applied over the M1 using a DS5 isolated bipolar constant current stimulator (Digitimer, Welwyn Garden City) connected via a cable to the input of a waveform generator (Peak Tech, Ahrensburg) (Chaieb et al., 2011). These existing devices cannot provide temporally interfering stimulation due to the weaknesses of the frequency range, channel number, and resolution, etc. Since the real modulating effect of time-interfering electrical stimulation on neurons is the modulating wave of two different kHz sinusoidal signals, this modulation process should not take place between the circuits of the equipment. In this work, high-quality current output was achieved using a reverse dual-current pump to restrain the crosstalk between channels.

The bioimpedance between electrodes during temporally interfering stimulation affects the actual distribution of the amplitude of the electrical stimulation. Theoretically, the electrical stimulation provided by the electric stimulator is a constant current source. However, due to the limited driving voltage, the biological impedance of each electric stimulation must be less than the limit load to ensure the signal stability of the constant current source, which is a feature of any electrical stimulation device. If the biological impedance between the two electrodes in one circuit exceeds the load limit, the actual current amplitude will be smaller than the preset current, and the target of the differential frequency stimulation will also be shifted toward this circuit current, as shown in the Supplementary Figure 3. In addition, if the bioimpedance is increased significantly during the electrical stimulation, this indicates that part of the electrode has bad contact, which will also affect the accuracy of the target location of the electrical stimulation, as shown in the Supplementary Figure 4. The bioimpedance measurement function can detect this condition early in the experiment and monitor the changes in impedance during the process of electrical stimulation in real time to ensure the effect of electrical stimulation. In the mouse experiment, we observed that the stimulation effect was better when the bioimpedance of each channel was small and not significantly different. In addition, bioimpedance decreased with an increase in the stimulation frequency in the common case (Rodriguez et al., 2016), indicating that the method of using a high-frequency current to modulate a low-frequency stimulation signal may be conducive to penetrating the barriers of the scalp, skull, and CSF.

In research on the mouse brain, the usual anchor point of the mouse head is a vertex on the skull called bregma. The bregma was also used as a reference point for the location of electrodes in the mouse electrical stimulation experiment in this study, so invasive treatment of the mouse scalp was required. However, the electrical stimulation itself is non-invasive, and in experiments on larger animals or humans, non-invasive methods can be used to stimulate the target. The following simulation results can be used as evidence. The simulation results shown in Supplementary Figure 5 show that the effects of non-invasive and invasive stimulation are similar.

Accurate stimulation of the target brain region is crucial for the application of electrical stimulation, and numerous studies on simulation localization for tDCS and tACS have been published (Datta et al., 2009; Edwards et al., 2013). In some studies, hundreds of electrodes are calculated to find optimal electrode configurations (Huang and Parra, 2019). The positioning accuracy depends on the use of a large number of electrodes, but only a few electrodes can be placed on the mouse skull, even if the electrodes are designed to be 1 mm in diameter. Based on the shape characteristics of the mouse head and brain, we found that the most effective point of stimulation always appeared on the same plane as the electrodes, so the three-dimensional individualized simulation navigation could be simplified into a columnar model simulation calculation according to the shape of the coronal plane. In this way, we could obtain electrode position prediction results by the functional relationship between the target coordinates, the electrode position and the amplitude ratio. Then, the target was further navigated in a three-dimensional, individualized model of the mouse, and the electrode positions were fine-tuned according to the simulation results.

Previous studies have shown that high-frequency electrical stimulation has an activating effect on neurons, which is based on its effect on cell membranes (Chaieb et al., 2011; Herrmann et al., 2013). The regulatory effect of low-frequency electrical stimulation on brain rhythms and networks has been recognized (Ali et al., 2013; Fröhlich, 2015), but kHz stimulation has shown no such effect. Time-interfering electrical stimulation, with the characteristics of both high-frequency and low-frequency electrical stimulation, may provide many opportunities for future research and is expected to more efficiently regulate brain function. Further studies, such as fMRI and behavioral experiments, will be meaningful and necessary for analyzing the changes in brain rhythms and networks derived from time-interfering electrical stimulation.

## Conclusion

In this study, we designed a temporal interference electrical stimulator in which technologies such as analog phase accumulation, reverse current pump driving, and spectral analysis were used to solve the problems of accurate output, crosstalk between channels, and bioimpedance measurements. The output performance of the device was confirmed by testing the load capacity and SNR of the device. A functional test of the bioimpedance measurement was performed under resistance and resistance-capacitance loads and in the mouse body. The uniformity of the measured and simulated distributions of the envelope amplitude exhibited the feasibility of temporally interfering electrical stimulation and the reliability of the stimulator. Through the simulation of idealized models and individualized mouse models, we achieved the precise positioning of temporally interfering stimulation targets. The functional relationship between the stimulus depth and the electrode spacing and the relationship between the target abscissa and amplitude ratio that we found can help users achieve target positioning in mice without modeling and simulation. Finally, we conducted a mouse experiment, and evoked movement was observed in the contralateral forepaw; that is, the temporally interfering stimulator succeeded in activating mouse neurons and achieved positioning accuracy in the mouse experiment. In summary, the performance and experimental effectiveness of the electrical stimulator have been verified in this study, and this type of stimulation, with both high-frequency and low-frequency electrical stimulation characteristics, provides many opportunities for future research. Further research should be carried out utilizing this electrical stimulator. If necessary, we will be able to provide equipment to research teams that need it.

## Data Availability Statement

The original contributions presented in the study are included in the article/Supplementary Materials, further inquiries can be directed to the corresponding author/s.

## Ethics Statement

The animal study was reviewed and approved by Ethics Committee of Beijing Tiantan Hospital affiliated to Capital Medical University, Beijing Tiantan Hospital.

## Author Contributions

HW, DC, and JWu contributed to the conception of the study. WS, JZ, and JWa performed the experiment. ZS and CL contributed significantly to the analysis and manuscript preparation. RY, YS, GG, and YX helped perform the analysis through constructive discussion. All authors contributed to the article and approved the submitted version.

## Conflict of Interest

YS was employed by the company Beijing Big-IQ Medical Equipment Co., Ltd. The remaining authors declare that the research was conducted in the absence of any commercial or financial relationships that could be construed as a potential conflict of interest.

## Supplementary Material

Supplementary Figure 1 Impedance measurement circuit. **(A)** Feedback signal transmission circuit. **(B)** Signal regulation circuit.

Supplementary Figure 2 Multichannel high-SNR output circuit.

Supplementary Figure 3 Influence of additional impedance on the target location.

Supplementary Figure 4 Influence of poor electrode contact on electrical stimulation. **(A)** There was complete contact between the electrodes and the mouse. **(B)** The condition in which a portion of the area between the electrodes and the mouse was in poor contact led to a decrease in the stimulus amplitude. **(C)** The condition in which a portion of the area between the electrodes and the mouse was in poor contact led to a right shift of the target.

Supplementary Figure 5 Comparison between non-invasive and invasive temporally interfering electrical stimulation. **(A)** Non-invasive model. **(B)** Invasive model. **(C)** Distribution of the stimulus intensity of the non-invasive model. **(D)** Distribution of the stimulus intensity of the invasive model.
