# Supplementary figures and images for: Development of a Non-invasive Deep Brain Stimulator With Precise Positioning and Real-Time Monitoring of Bioimpedance
Source: Front Neuroinform. 2020 Dec 8;14:574189. doi: 10.3389/fninf.2020.574189 (PMC7753039; doi:10.3389/fninf.2020.574189)

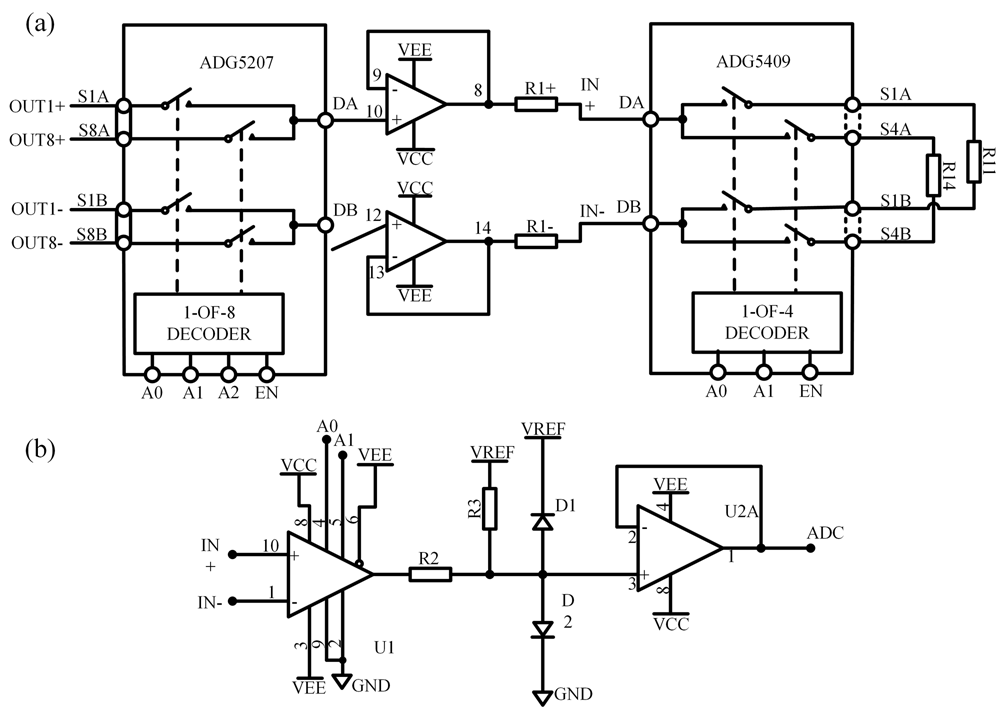

Supplement: Supplementary Figure 1 — Impedance measurement circuit. (A) Feedback signal transmission circuit. (B) Signal regulation circuit. [file Image_1.TIF]

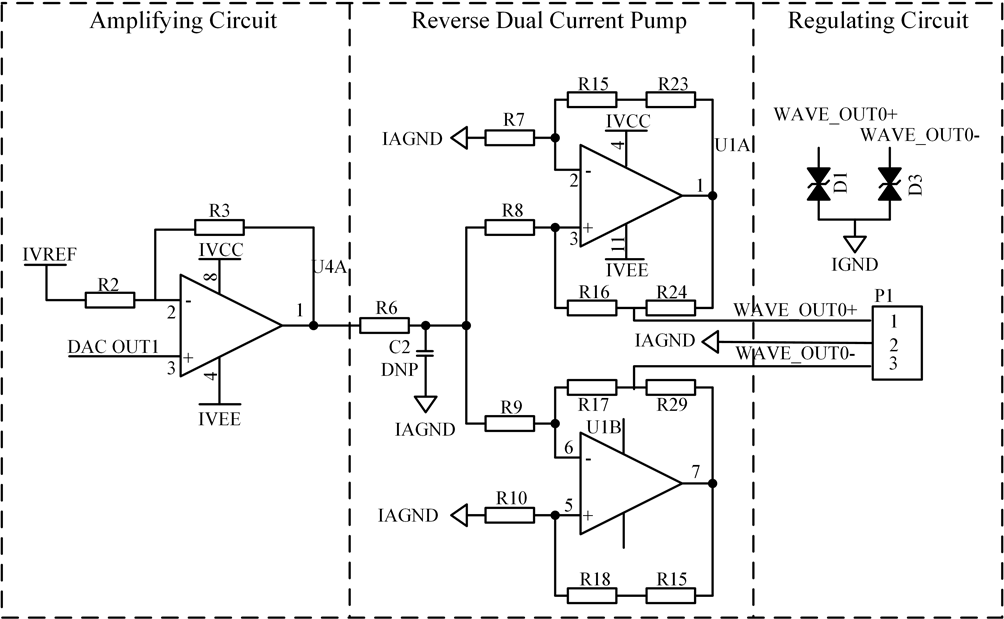

Supplement: Supplementary Figure 2 — Multichannel high-SNR output circuit. [file Image_2.TIF]

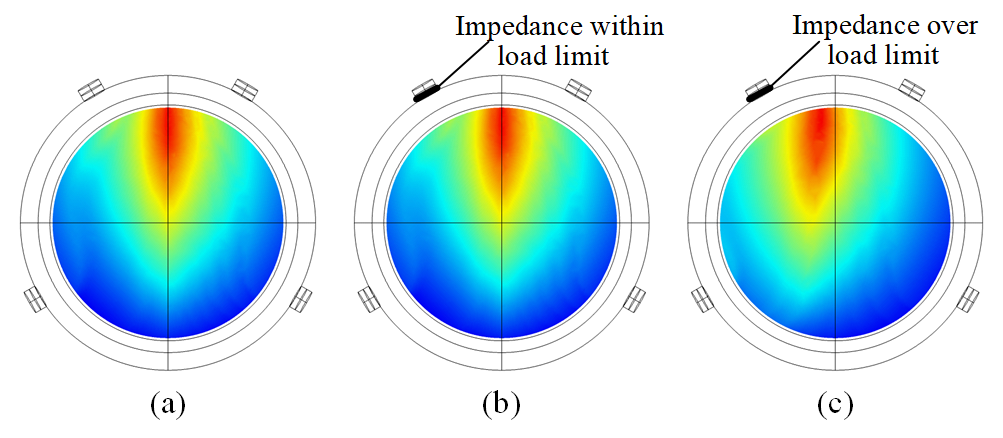

Supplement: Supplementary Figure 3 — Influence of additional impedance on the target location. [file Image_3.TIF]

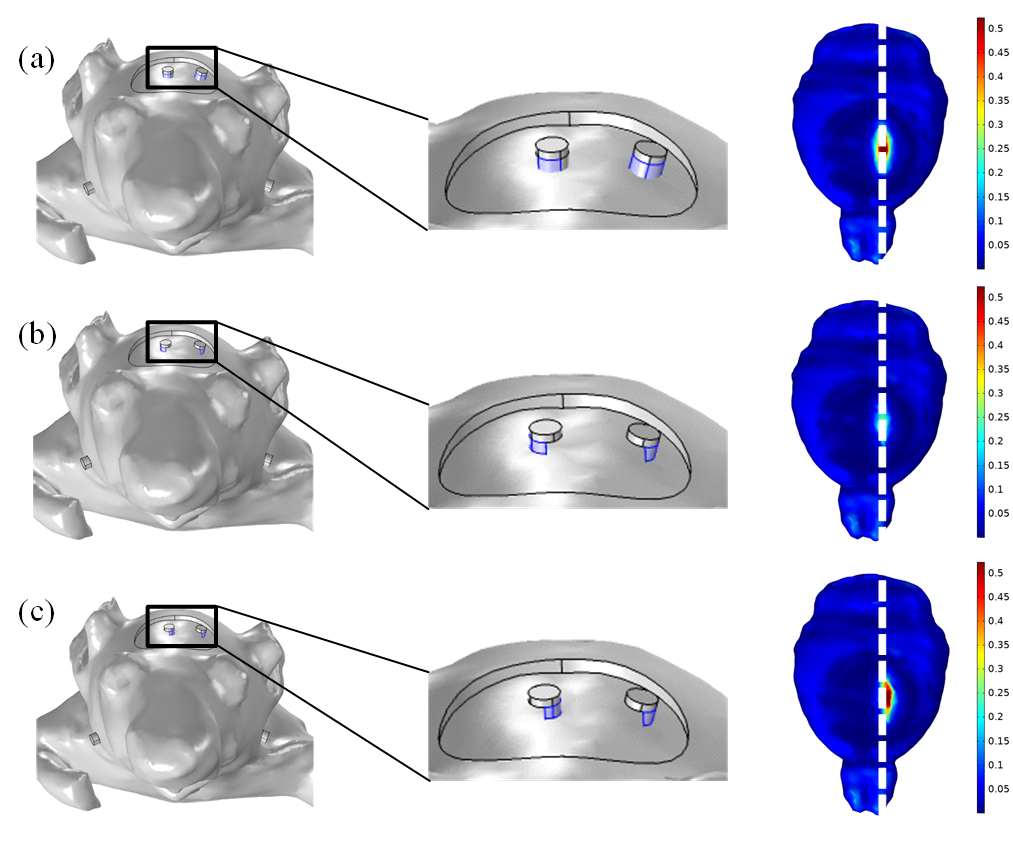

Supplement: Supplementary Figure 4 — Influence of poor electrode contact on electrical stimulation. (A) There was complete contact between the electrodes and the mouse. (B) The condition in which a portion of the area between the electrodes and the mouse was in poor contact led to a decrease in the stimulus amplitude. (C) The condition in which a portion of the area between the electrodes and the mouse was in poor contact led to a right shift of the target. [file Image_4.tif]

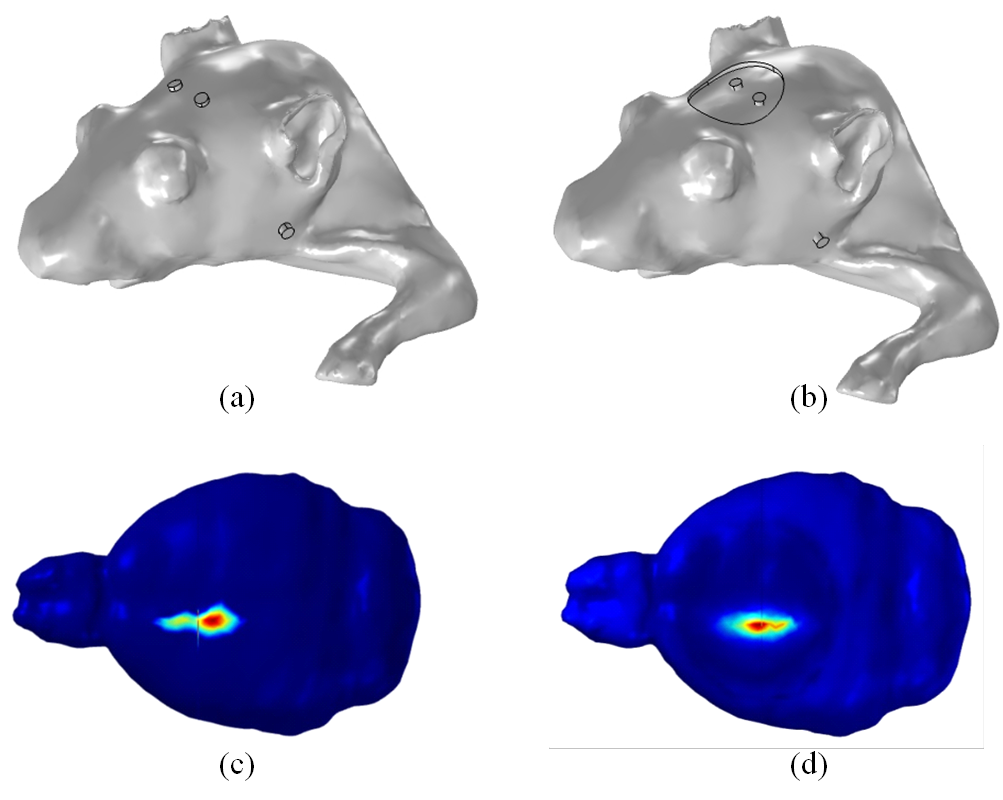

Supplement: Supplementary Figure 5 — Comparison between non-invasive and invasive temporally interfering electrical stimulation. (A) Non-invasive model. (B) Invasive model. (C) Distribution of the stimulus intensity of the non-invasive model. (D) Distribution of the stimulus intensity of the invasive model. [file Image_5.tif]
